# Frog skin as an alternative temporary dressing for diabetic foot ulcers treatment: animal model

**DOI:** 10.1590/acb406625

**Published:** 2025-08-18

**Authors:** Cristina Pires Camargo, Janayna Terlera Paulino, Deborah Luisa de Sousa Santos, Bianca Loula Santos, Ezequiel da Silva Passos Porto, Maria José Ferreira Alves, Miyuki Uno, Tatiane Katsue Furuya, Rolf Gemperli

**Affiliations:** 1 Universidade de São Paulo – Faculdade de Medicina – Microsurgery and Plastic Surgery Laboratory – São Paulo (SP) – Brazil.; 2 Universidade de São Paulo – Faculdade de Medicina – Multiprofessional Residency Program in Oncology Care for Adults – São Paulo (SP) – Brazil.; 3 Universidade de São Paulo – Faculdade de Medicina – Center for Translational Research in Oncology – São Paulo (SP) – Brazil.

**Keywords:** Diabetic Foot, Ulcer, Skin, Artificial, Heterografts

## Abstract

**Purpose:**

Diabetic foot ulcers (DFUs) are chronic lesions, and despite extensive research, no standardized treatment exists yet. This study aimed to evaluate the effect of frog skin application as a temporary dressing on the DFUs of diabetic rats.

**Methods:**

Diabetes was induced in male Wistar rats (n = 22) weighing 200–300 g through a streptozotocin injection (55 mg/kg). The animals received sub-doses of long-acting insulin to induce a moderate, chronic diabetic state. A DFU was surgically created on the animal’s paws (1 × 1 cm). The animals were divided into two groups: a control group treated with gauze and 0.9% saline solution (n = 10), and the frog skin group (n = 12). Both groups were further subdivided so it was possible to analyze their healing on postoperative 7 and 14 days (POD7 and POD14). The primary endpoints assessed included DFU contraction area, histological analysis, and gene expression.

**Results:**

On POD14, the frog skin group showed six times less contracture when compared to the control group. Histological analysis revealed a 30% increase in neoangiogenesis and 50% reduction in inflammation in the POD14 frog skin group when compared to the control group. Additionally, on POD7, *Il10* expression was about five times higher in the frog skin group compared to the respective control (*p* = 0.011).

**Conclusion:**

This study suggests that the use of frog skin as temporary biological dressing can reduce DFU secondary contraction and inflammation, offering potential therapeutic benefits for chronic wound management.

## Introduction

Diabetic foot ulcers (DFUs) are complex chronic wounds characterized by unique challenges such as vascular deficit, multi-bacterial colonization, and neuropathy. The literature has reported that 25% of patients with diabetes will develop DFUs^1^–^3^. Ulceration arises from a combination of neuropathy, circulatory dysfunction, and trauma, with prolonged wound exposure often leading to severe infection involving deep soft tissue layers^4^. Despite systemic glycemic control, the local DFU management remains a significant clinical challenge^3^,^5^,^6^.

The current care standard for DFU includes wound debridement, ischemia control, infection management, decompression, and the application of appropriate wound dressings to prepare the wound bed for reconstructive procedures^7^. However, DFU frequently requires multiple dressing and debridement sessions, prolonging the healing process^8^.

Wound dressings are widely used in DFU management due to their multifunctional benefits. Their properties are essential for promoting wound healing as they interact directly with the wound bed. Importantly, wound dressings must exhibit high biocompatibility, ensuring they do not cause toxicity or inflammation, and provide protection against infection^7^. A wide range of wound dressings is available, including organic alternatives. Notably, frog skin has been proposed as a potential dressing material, offering unique benefits such as lower immunogenicity compared to swine skin and the expression of antimicrobial proteins that disrupt bacterial membranes and inhibit proliferation^9^,^10^.

In this study, we evaluated the potential of frog skin as a temporary organic dressing for DFU therapy. We assessed DFU contraction area, histological changes, and relative gene expression in diabetic male rats one and two weeks after surgery to explore its therapeutic utility.

## Methods

Twenty-two male Wistar rats weighing 250–300 g were used in this study. The animals were housed individually in cages under a 12/12-h light/night cycle with food and water *ad libitum*. All experiments and procedures adhered to the local guidelines (Conselho Nacional de Controle de Experimentação Animal, 2013) and Animal Research: Reporting of In Vivo Experiments (ARRIVE) Guidelines. This study was approved by the Ethics Committee on the Use of Animals of the Medical School of the Universidade de São Paulo, under registration no. 1,061/2018.

### Moderate chronic diabetes induction

The animals were anesthetized with isoflurane (100–150 mL/min, Cristália, Itapira, SP, Brazil). Following antisepsis, streptozotocin 1% streptozotocin (STZ) mixed anomers (031M1287V, Sigma Chemical, St. Louis, MO, United States of America) diluted in phosphate buffered saline (PBS) (pH = 4,5) was injected via the penile vein according to animal weight (55 mg/kg). Blood samples were collected from the tail 24 hours post-induction, and glycemia was measured using a One Touch glucometer (LifeScan, Johnson & Johnson, Malvern, Pennsylvania, United States of America). Animals with blood glucose levels greater than 200 mg/dL were classified as diabetic, and all animals met this criterion.

Additionally, 15 days after the induction procedure, 1–2 IU of Lantus insulin (Sanofi-Aventis, Bridgewater, New Jersey, United States of America) was administered subcutaneously in the dorsal compartment twice weekly at 4 p.m. for two months^11^. Blood glucose levels were monitored weekly for eventual insulin dosage adjustment. Chronic moderate diabetes was defined as blood glucose levels maintained between 300–350 mg/dL12.

### Frog skin organic dressing production

Frog skin was obtained from a commercial vendor and prepared under sterile conditions. It was initially cleaned using a 0.9% saline solution (Baxter, São Paulo, SP, Brazil) and then immersed in a 99% glycerin solution (Porto Alegre, RS, Brazil) for three days. Then, the frog skin was washed with 0.9% saline solution and stored in 50% glycerol solution until the day of the surgery. To ensure sterility, a Gram stain was performed to detect potential bacteria contamination. Thirty minutes prior to the surgery, the frog skin was hydrated in a 0.9% saline solution.

### Surgical procedure

All animals were anesthetized via intraperitoneal injection of ketamine hydrochloride (100 mg/kg, Cristália, Itapira, SP, Brazil) combined with xylazine hydrochloride (5 mg/kg, Bayer, São Paulo, SP, Brazil). Chlorhexidine (Rioquímica, São José do Rio Preto, SP, Brazil) was used as the antiseptic. A square wound (1 × 1 cm) was surgically created on both anterior paws of each animal using a #15 scalpel.

The animals were then randomized into two groups: control (n = 10) and frog skin (n = 12). In the control group, the wounds were dressed in gauze soaked in saline solution, with dressings changed every two days. In the frog skin group, the wounds were covered with frog skin, which was fixed with a 4-0 polyimide suture (Mononylon, Johnson & Johnson, Malvern, Pennsylvania, United States of America). After applying the dressing, each group was further divided into two postoperative time points: seven days (POD7) and 14 days (POD14).

The primary outcome was the percentage of DFU contracture, and the secondary endpoints were microscopic and relative gene expression analyses at POD7 and POD14.

### Macroscopic analysis

Wounds were photographed alongside a ruler (mm scale) to ensure accurate measurement. The images were then transferred and imported into the ImageJ Pro software (NIH, Bethesda, MD, United States of America). Following focus calibration to the rules for scale, the software was used to accurately measure the DFU area.

### Microscopic analysis

All samples were fixed in 4% formalin for 24 hours and subsequently embedded in paraffin for hematoxylin-eosin (HE) staining. Histological analyses were performed under 200x and 400x magnification using an optical microscope (Nikon Eclipse E600, Tokyo, Japan).

Quantification of histological structures was performed in ten randomly selected fields per sample. The endpoints analyzed with HE staining included the presence of dermic appendages, arteriole count (angiogenesis), and inflammatory cell count. All samples were analyzed blindly to ensure objectivity.

### Gene expression analysis of wound healing markers

Total RNA extraction (30 mg) was performed using the RNeasy Mini Kit (Qiagen, Germantown, United States of America), as described previously by our group^13^. RNA purity was evaluated through the ratios 260/280 and 260/230 by spectrophotometry (NanoDrop^TM^ 1000 Spectrophotometer, Thermo Fisher Scientific, Waltham, MA, United States of America), and RNA integrity was determined by gel electrophoresis. RNA samples were stored at -80°C until use.

For quantitative reverse transcription polymerase chain reaction (qRT-PCR), total RNA was reverse transcribed into cDNA using the High-Capacity RNA-to-cDNA Kit (Thermo Fisher Scientific, Waltham, MA, United States of America) following the manufacturer’s instructions. Gene expression levels of *Cd68* (Rn01495634_g1), *Il10* (Rn01483988_g1), *Il1rap* (Rn01404183_m1), *Mmp3* (Rn01495634_g1), *Mmp9* (Rn00579162_m1), and *Tnf* (Rn01525859_g1) were quantified using TaqManTM methodology (Thermo Fisher Scientific, Waltham, MA, United States of America) on the StepOnePlus Real-Time PCR system (Thermo Fisher Scientific, Waltham, MA, United States of America), under the default cycling conditions specified by the manufacturer. Each sample was run in duplicate, with *Gapdh* (Rn01775763_g1) and *Actb* (Rn00667869_m1) genes used as endogenous controls. Relative gene expression was calculated using the comparative CT method (2^−ΔΔCT^)^14^.

### Statistical analysis

The sample size calculation was based on a pilot study (data not shown) involving five animals per group. This pilot study indicated that the wound area contraction was 80% in the placebo group and 20% in the frog skin group. Using this data, the required sample size was calculated with the Cochran-Armitage test, resulting in ten animals per group. Parameters included a W alpha of 5% and a power of 80%, calculated using STATA v18 (StataCorp, College Station, TX, United States of America). Due to the small sample size, data analysis was performed using nonparametric tests, and results are shown as medians with interquartile intervals. Gene expression analysis was performed using Statistical Package for the Social Sciences version 25 software, separately for the POD7 and POD14 experiments. Comparisons of -dCT values between the studied groups were conducted using the nonparametric Mann-Whitney’s test. The control group was considered as reference for all comparisons.

## Results

No infections or adverse events were observed during this study.

### Macroscopic analysis

The contracture ratio analysis revealed that on POD7, the DFU area in the frog skin group was 70% greater than in the control group (55.6 vs. 32.8, respectively; *p* = 0.009). On POD14, the frog skin group exhibited six times less contracture when compared to the control group (43.3 vs. 7.5, respectively; *p* = 0.008), as shown in Table 1.

**Table 1:** Diabetic foot ulcer areas in the control and frog skin groups on postoperative day 7 (POD7) and 14 (POD14) are presented as median (interquartile interval).

	Control POD7	Frog skin POD7	Control POD14	Frog skin POD14
**Area (mm^2^)**	32.8 (26.5–44.2)	55.6 (41.9–65.7)*	7.5 (4.2–8.2)	43.3 (40.0–50.3)**

* *p* = 0.009 vs. control POD7;

** *p* = 0.008 *vs*. control POD14.

Source: Elaborated by the authors.

### Microscopic analysis

**Figure 1:**
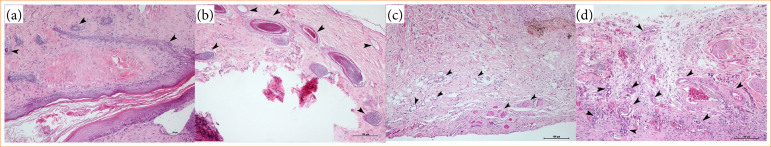
Representative photographs of diabetic foot ulcer skin stained with hematoxylin-eosin of the groups: **(a)** control postoperative day 7 (POD7); **(b)** frog skin POD7; **(c)** control postoperative day 14 (POD14); **(d)** frog skin POD14. Arrows indicate blood vessels. Magnification 200x. bars represent 100 µm.

Source: Elaborated by the authors.

Figure 1 and Table 2 show the results of the microscopic analysis. On POD7, no significant difference was observed between the groups regarding dermal appendages. However, on POD14, the frog skin group exhibited about 60% increase in dermal appendages compared to the control group (44 *vs*. 28, respectively; *p* = 0.03). Additionally, arteriole counts (neoangiogenesis) were about 30% higher in the frog skin group compared to the control group on both POD7 (*p* = 0.003) and POD14 (*p* = 0.002). Regarding inflammatory cell counts, although no significant, the frog skin group showed about 10% reduction of cells on POD7 (*p* = 0.07) and a significant 50% reduction on POD14 (*p* = 0.0002) compared to the control group.

**Table 2:** Microscopic analysis of dermic appendages, arterioles count, and inflammatory cells counts in the control and frog skin groups at postoperative day 7 (POD7) and 14 (POD14). Data are presented as median (interquartile interval).

	Control POD7	Frog skin POD7	Control POD14	Frog skin POD14
Dermic appendages	12 (8–14)	21 (15–22)	28 (25–44)	44 (33–47)*
Arteriole	10 (9–12)	17 (11–18)**	10 (8–12)	18 (15–21)***
Inflammatory cells	108 (103–129)	94 (83–101)#	141 (122–154)	68 (66–74)$

* *p* = 0.03 vs. control POD14;

** *p* = 0.003 vs. control POD7;

*** *p* = 0.002 vs. control POD14;

# *p* = 0.07 vs. control POD7;

$ *p* = 0.0002 vs. control POD14.

Source: Elaborated by the authors.

### Gene expression

Table 3 summarizes the gene expression results for POD7. On POD7, the *Il10* expression levels were about five-fold higher in the frog skin group when compared to the control group (*p* = 0.011). The results for POD14 are presented in Table 4. No significant differences in gene expression levels were observed between the groups.

**Table 3:** Gene expression profiles of control (n = 5) and frog skin (n = 6) groups on postoperative day 7 (POD7) are presented as mean ± standard deviation of fold change.

Genes	Control POD7 FC (mean ± SD)	Frog skin POD7 FC (mean ± SD)	*p* -value
*Cd68*	1.06 ± 0.37	0.82 ± 0.18	0.201
*Il10*	1.05 ± 0.31	5.69 ± 2.77	0.011*
*Il1rap*	1.04 ± 0.21	0.97 ± 0.26	0.584
*Mmp3*	1.75 ± 2.31	3.57 ± 5.03	0.855
*Mmp9*	1.24 ± 0.80	0.56 ± 0.49	0.144
*Tnf*	1.05 ± 0.36	1.72 ± 1.07	0.273

FC: fold change; SD: standard deviation; Mann-Whitney test;

* *p* < 0.05.

Source: Elaborated by the authors.

**Table 4:** Gene expression profiles of Control (n=5) and Frog Skin (n=6) groups on postoperative day 14 (POD14) are presented as mean ± SD of fold change.

Genes	Control POD14 FC (mean ± SD)	Frog skin POD14 FC (mean ± SD)	*p* -value
*Cd68*	1.04 ± 0.34	0.89 ± 0.51	0.522
*Il10*	1.32 ± 1.12	1.44 ± 0.78	0.831
*Il1rap*	1.03 ± 1.09	1.23 ± 0.25	0.394
*Mmp3*	2.42 ± 1.77	4.68 ± 2.94	0.286
*Mmp9*	1.42 ± 1.20	3.33 ± 2.33	0.136
*Tnf*	1.05 ± 1.11	1.74 ± 1.53	0.286

FC: fold change; SD: standard deviation; Mann-Whitney test. Source: Elaborated by the authors.

## Discussion

According to the deformation field theory of scar formation, a skin wound requires a mechanical field to counteract linear forces that lead to contraction, balancing collagen production and degradation^15^. The linear tension forces activate myofibroblasts, promoting fibrosis and secondary wound skin contraction^15^,^16^. Several studies have evaluated the use of scaffolds and dermal substitutes to model secondary wound skin contraction^15^–^18^.

In the present study, we assessed the utility of decellularized frog skin as a biological scaffold and showed some benefits. We hypothesized that frog skin attenuates linear tension and inflammation associated with DFUs. The secondary contraction can cause pathological fibrosis, impairing tissue function in areas such as cervical and articular regions. Pathological fibrosis also results in dysfunctional tissue scars that negatively affect the reconstructive procedure^19^. Therefore, a therapeutic approach to reduce local tissue fibrosis would be desirable in clinical practice.

Previous studies have proposed the use of epidermal or dermal substitutes (biological, synthetic, or biosynthetic) as treatment strategy for wounds^20^,^21^. Biological temporary dermal substitutes, such as porcine skin, pose a significant risk of high host-graft immunogenicity, leading to their abandonment in daily clinical practice^22^. While synthetic dermic/epidermic substitutes offer advantages, their high costs often make them impractical, especially in low-middle income countries^23^,^24^. Recently, several studies have analyzed the use of tilapia (*Oreochromis niloticus*) skin as a temporary biological substitute for burn injuries. Most studies have reported promising results on the effects of tilapia skin on burn wounds, but there is limited data on the wound contraction effect. Furthermore, there are no studies addressing its use on infected or potentially infected wounds^25^,^26^.

Another temporary biological substitute that has been evaluated is frog skin. Several studies have highlighted the presence of antimicrobial peptides (AMP) in frog skin, making this tissue a promising dressing for chronically infected wounds. AMPs are short chains of amino acids with the ability to eliminate microorganisms. To date, around 2,600 AMPs have been catalogued, including some with immunoregulatory properties^27^. The bactericidal action of AMPs is largely attributed to their cationic ability, with 88% of these peptides being positively charged. This positive charge allows AMPs to interact with the anionic bacterial plasma membrane, leading to the disruption of the membrane and the extravasation of the microorganism’s cytosol through the toroidal pore mechanism^28^.

In our study, the application of frog skin dressing effectively preserved the DFU area by reducing the wound contraction, resulting in 50% more wound area on POD14 compared to the control group. Histological analyses demonstrated a 30% increase in neoangiogenesis and a 60% increase in the number of dermal appendages in the frog skin group relative to the control group on POD14. Hypoxia is a major factor hindering diabetic wound healing. Hypoxia is a key factor impairing diabetic wound healing, as oxygen is essential for regulating critical healing processes. By enhancing blood flow and re-epithelialization, as indicated in our study by an increase in the number of blood vessels and dermal appendages, frog skin dressing can create a more favorable environment for successful skin grafts29.

Furthermore, the study showed a significant 50% reduction in inflammatory cell count in the frog skin group when compared to the control group on POD14. Additionally, the *Il10* expression levels were about five-fold higher in the frog skin group when compared to the control group on POD7. These findings indicate that frog skin dressing does not exacerbate inflammation at the wound site and may actively reduce inflammation in DFU. The reduction in inflammatory biomarkers directly improves collagen remodeling, preventing pathological fibrosis^30^, thereby promoting more effective and functional wound healing.

Skin grafts often fail when applied to wound beds with insufficient blood supply, bacterial infection, or a high inflammatory response. Therefore, proper preparation of the wound bed is crucial for the success of skin grafts9. The use of frog skin may offer a promising alternative to improve the outcomes of these surgical procedures, as temporary frog skin dressing showed effectiveness in maintaining a viable wound, with less contraction and with increased angiogenesis to sustain a later skin graft.

Although our study has some limitations, such as using a murine model with anatomical and histological differences from human skin and a small sample size due to the experimental nature of this analysis, it is the first one to evaluate the efficacy of frog skin in reducing chronic wound contraction.

## Conclusion

This study highlights the potential benefits of using frog skin dressings as temporary biological dressing for DFU, particularly for their ability to reduce secondary skin contraction and inflammation. Further research should be necessary to explore the antimicrobial properties of frog skin to fully assess its therapeutic potential in chronic wound care.

## Data Availability

The data will be available upon request.
